# Responsibility for patient care in perioperative practice

**DOI:** 10.1002/nop2.153

**Published:** 2018-04-27

**Authors:** Ann‐Catrin Blomberg, Birgitta Bisholt, Lillemor Lindwall

**Affiliations:** ^1^ Department of Health Sciences Karlstad University Karlstad Sweden

**Keywords:** ethic, hermeneutic text interpretation, operating theatre nurse, perioperative nursing, responsibility

## Abstract

**Aim:**

To obtain an understanding of operating theatre nurses' experiences of responsibility for patient care and safety in perioperative practice.

**Design:**

A hermeneutic design were used.

**Method:**

Data were collected during 2012 from 15 operating theatre nurses who participated in individual interviews. The text was analyzed by hermeneutical text interpretation.

**Findings:**

The texts revealed two main themes: A formal external responsibility and personal ethical value. Responsibility that the patient was not exposed to risks, protecting the patient's body, systematically planning and organizing work in the surgical team. The personal ethical value meant confirming the patient as a person, caring for the patient and preserving the patient's dignity. A new understanding emerged that the operating theatre nurse always have the patient in mind.

## 1 INTRODUCTION

In this study, we want to obtain an understanding of the operating theatre nurses' (OTNs') responsibility for patient safety and care in perioperative practice. Perioperative practice is demanding; it is complex, highly technical and very different compared with other settings. OTNs are responsible for aseptic, instrumentation, infection prevention and complication measures, medical technique, handling of biological preparations, as well as collaborating and planning care in consultation with the patient, surgical team and other healthcare providers (Gillespie & Hamlin, 2009; Kelvered, Öhlén, & Gustafsson, 2012). OTN's often work in challenging situations under a fast pace and have to address the basic concepts that are unique in ensuring the surgical patient's safety (Spruce, 2013).

Perioperative nursing was introduced in the US by Association Of perioperative Registered Nurses (AORN) (1978) as nursing activities performed during the pre‐, intra‐ and postoperative phase of a patient's surgery. Lindwall and von Post (2008) described perioperative nursing in relation to Swedish conditions and the text was revised by Lindwall and Blomberg (2018) as: “Perioperative nursing is a nurse anaesthetists” and operating theatre nurses' pre‐, intra‐ and postoperative care for a patient who is undergoing surgery. Perioperative nursing includes all nursing activities related to the surgical treatments, organization and leadership of the perioperative practice. Perioperative dialogues are nurse anaesthetists' and operating theatre nurse's pre‐, intra‐ and postoperative dialogues with the patient, with the purpose to plan, implement and evaluate perioperative nursing and create continuity in patient care'.

Association Of perioperative Registered Nurses (AORN) (2015) continuously develops standards of responsibility and safety for the patient's care, where the OTN needs to exercise judgement based on education and experiences to determine what appropriate care is for the patient based on evidence. According to Bull and FitzGerald (2006) professional competence is perceived as medical technology oriented. Sørensen, Olsen, Tewes, and Uhrenfeldt (2014) highlights the relationship between nursing and technology and this involves a responsibility for patients' care. Alfredsdottir and Bjornsdottir (2008) also emphasize responsibility towards the organization's current production requirements that implicitly affect the patient's care. This explains the interest in investigating how OTNs experience their responsibility in perioperative practice.

## 2 BACKGROUND

Each Registered Nurse (RN) has ethical and moral responsibility to represent the patient's interests, show humility, respect and protect patient autonomy and preserve patient dignity (International Council for Nurses, 2013). Responsibility according to Wallinvirta (2011) is a universal term and relates to how OTN's nursing activities are made visualized in patient care, but also to fulfil a fundamental duty for the other, thus constituting an ethical requirement and a duty to the other and to oneself. The relationship between nurse and patient is asymmetric and according to Karnick (2016) it is important to know that patients have their own power of self‐determination and that it is the nurse's responsibility to have an understanding of it in a mutual community. Lévinas, Nemo, and Contassot (1988) ethics means that by seeing the other's face I become responsible and committed to the other in a vulnerable situation. Clancy and Svensson (2007) empasized that, based on Levinas ethical theory, the nurse's understanding was deepened, to be responsive not only for the predictable but also dare to face unpredictable fear, concern and insecurity.

Responsibility has different meanings, an external responsibility, which includes principles, guidelines and practical rules and an overall responsibility towards organization and society, assuming legal and moral rights. Internal responsibility means an ethical attitude about own responsibility, values and the will to do good (Wallinvirta, 2011). Sjögren (2012) and von Post (2000) mean that an interpersonal meeting is an ethical dimension and implies an external responsibility in the form of the duty of professional responsibility, which usually does not require a deeper personal position. However, internal responsibility requires a personal attitude and courage to meet and create a relationship with the other and become concerned as a human being. It also includes taking care of yourself and your personal development, to be responsible for the other. OTNs are faced with different personal positions regarding how to perform patient care in a dignified way.

Previous research has focused on OTN's responsibility related to cooperation in the surgical team and the surgeon intraoperatively (Björn & Boström, 2008; Mitchell et al., 2011). Having personal knowledge about the surgeon's wishes and requirements was important for the surgery implementation according to Riley and Manias (2006) and Sandelin and Gustafsson (2015) emphasized the importance of mutual cooperation and trust in the knowledge of the surgical team. Gillespie, Chaboyer, Wallis, Chang, and Werder (2009a) emphasized the importance of communicating and taking responsibility for coordinating and organizing work in the surgical team through theoretical, practical, situational and aesthetic knowledge for the implementation of the operating list. Kelvered et al. (2012) describe that the OTNs responsibility is to retrieve information from and about the patient, which influence planning prior to surgery and help control the situation and increase preparedness for unforeseen events. Ingvarsdottir and Halldorsdottir (2017) conducted interviews with experienced OTNs that showed how they took responsibility for the patient's vulnerability and safely navigated the patient through the nursing process. Arakelian, Swenne, Lindberg, Rudolfsson, and Vogelsang (2016) study emerged that the patients perceived that they were respected as a person, taken seriously and participated in their care if it was carried out in a person‐centred manner. On the other hand, Gillespie and Pearson (2013) showed that the OTN took responsibility for “caring skills”, which was not shown by the surgical technicians. Because of uncertainties in the OTNs responsibility for the patient, the aim of this study was to obtain an understanding of OTN's experiences of responsibility for patients' care and safety in perioperative practice.

## 3 THE STUDY

### 3.1 Design

In this study we chose a hermeneutical approach inspired by Gadamer's (1989) philosophy. A hermeneutical study implies that interpretation and understanding of the data obtained is based on the researchers' existing knowledge and experience in the subject. Hermeneutic text interpretation seeks to understand the substance of the text before declaring the speaker behind the text.

### 3.2 Participants

The participants consisted of 15 strategically selected OTNs (14 women and one man), 34–60 years, professional experience in perioperative practice ranged from 6–36 years. The inclusion criteria were OTNs having at least 3 years professional experience in perioperative practice, both women and men and place of employment at university, county/regional and district hospitals in the middle of Sweden. The participants were RN and had different educational backgrounds. Half of the participants had a direct education to become an OTN while others had postgraduate education in theatre care. Only three participants had an academic degree.

### 3.3 Data collection

The data were collected through individual interviews (Kvale, Brinkmann, & Torhell, 2009). A request for participation was sent by post to the head of operating departments. After approval for participation, nurse managers were contacted by e‐mail, who asked prospective participants and forwarded their names and e‐mail. The first author contacted the participants and conducted interviews in a schedule room in the operating theatre, which lasted between 45–60 min. The interviews were digitally recorded and transcribed verbatim. The data collection was carried out during autumn 2012.

### 3.4 Hermeneutic text interpretation

The transcribed texts from interviews were subjected to hermeneutical text analysis to gain an understanding of OTN experiences of responsibility. Gadamer (1989) focused on pre‐judgement, pre‐understanding and fusions of horizon and emphasized that those who understand are connected by a common human consciousness that makes understanding possible. Interpretation of the interviews was initiated with a review of the text as the original source and its relevance in practice.

#### 3.4.1 The interpretation of the interviews was done in five steps:

##### The first reading—integrating the text with the reader

It began as an open reading, which meant that the reader (ACB) asked the text about responsibility in perioperative practice. The interpretation of the texts was influenced by caring science as well as knowledge and clinical experience as an OTN in perioperative practice. The text was read from beginning to end, because the last part can change everything and each reading is a translation (Gadamer, 1989). When reading the text questions emerged such as: Is this the OTNs' responsibility in perioperative practice? The text answered: Yes, they described it like that.

##### The second reading—fusion of horizons

The interviews were read carefully which allowed them to present all their otherness and be a part of the reader. Professional pre‐understanding had been taken in account in relation to content that was unfamiliar and new questions emerged: How do the OTNs experience their responsibility in perioperative practice. Gadamer (1989) stated that when our horizon of understanding meets another horizon of understanding, a fusion of horizon occurs, which alters our understanding and a new world opens up for us. In this step, it became apparent that the text had a new understanding.

##### The third reading—new questions to the text

The following questions arose when the researcher transcended the horizon of the text: What do OTNs perceive as responsibility in *perioperative practice*? The text was studied further to find answers to the question. Searching for quotations in the text with common and distinguishing qualities, movements back and forth throughout the text for significant expressions.

##### The fourth—summarizing the main themes and subthemes

The text was carefully read through to search for common features in all significant expressions. The common features were formed into two main themes with associated subthemes. Each subtheme received its design using quotes from the original text.

##### The fifth—new understanding

**Figure 1:**
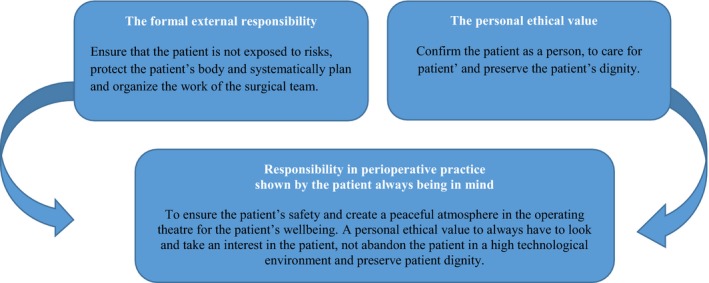
Responsibility of the operating theatre nurses in perioperative practice

The entire text was read again to reconfirm the themes and the emerging new understanding by moving back and forth between the parts and the whole. This process of understanding involved abstraction of the main and subthemes to from a coherent completely (Figure 1).

### 3.5 Ethical consideration

The study followed ethical principles in accordance with the Helsinki Declaration (2013), protecting the research subjects' anonymity, privacy and maintaining public confidence. The study was also approved by the local university ethics committee (Dnr C2012/306.) Written informed consent was obtained from all participants and documented according to the law for ethics review (SFS 2003).

## 4 RESULTS

The results revealed the OTN's experiences of responsibility for patient's care in perioperative practice as two main themes: “the formal external responsibility” and “personal ethical value”.

The first main theme, formal external responsibility consists of three subthemes: “Ensure that the patient is not exposed to risks, protect the patient's body and systematically plan and organize the work in the surgical team”.

### 4.1 Ensure that the patient is not exposed to risks

The formal external responsibility is the duty that all material and equipment is prepared and controlled before surgery. Current data are collected from the patient prior to planning the patient's pre‐operative preparation. The patient's identity is checked to ensure that the surgery is performed on the correct side when operating on paired organs. All preparations must be completed before the patient arrives in the operating theatre with the intention of creating a calm and familiar environment:

It is for the patient that I prepare with the right instruments and materials. Functional control of equipment and if the patient is overweight or has allergies or is treated with cortison… I must consideration of the choice of sterile draping, dressing and choice of instruments… Patients who are not conscious, I check the anaesthesia record and if the patient is awake we check the checklist. It is so calm so, if everything is done before the patient enters the operating theatre. (#10)

Some participants felt extremely responsible for the preparation of the patient's positioning on the operating table, that the right extra equipment was adapted according to the patient's problems and needs as well as the current surgical technique. A responsibility was also to make sure that the patient was informed of what was planned. OTNs know from the knowledge of surgery techniques and professional experience in perioperative practice, which pressure and nerve damage can occur during positioning and can answer the patient's questions in this situation. Before surgery, the patient's positioning is controlled to ensure that the patient is not exposed to risks:

It is my responsibility to choose the right operating table with accessories because we know what is expected and some surgery require special X‐ray tables and they must be from the beginning… If the surgery requires more space, I need to know where the surgeon and patient's armrest are best placed. From experience we know where pressure can occur…its also up to me to find out that the patient is aware of what is planned. (#5)

To ensure the patient safety, OTNs are responsibility based on knowledge of topographical anatomy and surgery methodology for everything being prepared and controlled and the patient's positioning is optimal.

### 4.2 To protect the patient's body

OTNs' responsibility includes protecting the patient's body. The exposed and vulnerable situation requires that he/she is vigilant that the patient's body is not exposed to discomfort and suffering. Responsibility also means preventing hypothermia, especially in the care of elderly patients:

… I always cover the patient to prevent the patient from being exposed or getting cold… even if it is an acute patient. If intimate parts have been exposed, we lower the legs and cover with a sheet before surgery is finished. I raise the temperature of the operating theatre to maintain body temperature… when the patient is protected with an electric blanket, the temperature can be lowered… but not if it is an older person who needs extra warmth. (#13)

During the intraoperative phase, the OTN protect the patient's body from pressure and nerve damage. He/she takes responsibility for continuously performing various relieving exercises and checks that the bedding is dry and smooth under the patient. They prevent and notice if someone in the surgical team exposes the patient to pressure on parts of the body. If an extreme position is required during the surgery, the OTN takes responsible for restoring the patient's body position, when no longer needed:

… To continuously “scan” the patient's body during the draping… see that the patient is in a comfortable position, that it does not leak, the draping is tight and that no one stands and press on the patient. Raise and lower the leg supports once every half an hour, touch the legs slightly and massage the feet. If the patient is in an extreme position, the surgeon is told that the patient may be injured (#7)

Most OTNs described their responsibility to be prepared for unforeseen events. To keep order and be concentrated on the surgical wound as well as preparing for the next step which may affect the patient's surgical time. OTNs continuously checking all materials used and postoperatively ensure that nothing is left in the patient:

I want to fix the instruments if I need something quickly… is concentrated on the wound if something occurs… to be one‐step ahead. Before the surgery ends and suturation begins, focus is on all material being controlled and ensuring that nothing remains in the patient's body. (#15)

The responsibility is to prevent the patient from being exposed to risks. It includes protecting the patient's body, respecting the patient's integrity, preventing hypothermia and pressure and nerve damage. To be prepared for unforeseen events and postoperatively ensure that nothing remains in patient's body.

### 4.3 Systematically plan and organize work in the surgical team

When the patient's positioning is not optimal, there is a risk that sterility is compromised or when problems arise related to medical equipment, the OTN takes responsibility and assumes a supervisory function in the surgical team. Responsibility for the patient means preventing postoperative infections by minimizing the number of people and flow in the operating theatre:

… when the patient's position is wrong, or something becomes unsterile… I control unnecessary traffic through the door… and not too many in the operating theatre because the risk of infection increases if the ventilation is not working… or questions regarding medical equipment… then I take responsibility and control the situation with a supervisory function. (#15)

Collaboration in perioperative practice means that all in the surgical team work efficiently in implementing the operating list, to minimize patient surgical time. Often, the OTN coordinates and organizes work in the surgical team and in acute situations clearly prioritizes different nursing activities:

We have a program that we jointly implement. We need to have a completed and clear plan for it to flow… if I see that they are doing something that has to be done but not right now… they must do it in the correct order. In acute surgery it is important prioritize. (#1)

The OTN formal external responsibility are to ensure the patient's positioning on the operating table and prevent postoperative infections in the surgical team. It also includes organizing of the surgical work and in acute situations clearly prioritize different nursing activities.

The second main theme, personal ethical value, consists of four subthemes: “confirm the patient as a person”; “to feel in and be responsive in the situation”; “to care for the patient” and “to preserve the patient’s dignity”. [Correction added on 22 May 2018, after first online publication: the last subtheme was missing and has been added]

### 4.4 Confirm the patient as a person

To really see the patient as a human being and not to objectify the patient is described by OTNs as a personal ethical value in the care of the patient. Showing humanity through a glance at a brief contact can contribute to a mutual community with the patient:

To really see and confirm the patient's presence… and don't just see a stomach because it's easy in this job… important to be involved when the patient is laid up for just a glance during a brief contact can create a relationship… to see a face who will be included in the surgery to recognize me… much can be conveyed through body language, touch and eye contact. (#12)

A personal ethical responsibility is to see, confirmed the patient and create a mutual community.

### 4.5 To feel in and be responsive in the situation

The personal ethical value includes being present in the patient's care. Some OTNs described experiences that the patient often expresses a sense of vulnerability before surgery:

… I have experience from the surgical ward that patients are often frightened before surgery. One thing is that you lose control of your body… lying on a bunk… then you have immediately lost your identity and are in a state of subversion. (#5)

To be sensitive to what the patient wants to convey in the situation, described OTNs as a personal ethical responsibility and a characteristic developed through professional experience. It is important to understand that the caring relationship is asymmetrical:

…from patient to patient to feel in and “read”… if they are stressed and nervous. Some want to talk while others are silent… others want to be concerned and others not… then the surgical team have to respect that and not push on. (#15)

Personal ethical values is expressed by being present and responsive in the situation as well as understanding that the relationship is asymmetrical.

### 4.6 To care for the patient

A common responsibility in the surgical team is to show that they care for the patient and do not abandon the patient for practical things but always have an interest in the patient. Intra‐operatively, the OTN's patient relies on how he/she experiences the situation by continuously saying what is going on:

In the surgical team, we constantly look at the patient so that they feel well. We always try to explain what is happening… if the patient is awake. I stick my head over the anaesthetic arc and ask if he/she feels well…. Some say nothing, but they look and listen to everything. (#6)

A common responsibility in the surgical team is to care for and not abandon the patient. The OTN constantly keeps a watchful eye on the patient.

### 4.7 To preserved patient's dignity

Maintaining patient dignity is a personal ethical value. OTNs are affected when unnecessary conversations are entered into the operating theatre and the patient's dignity is not preserved. If a conversation is to be conducted, it should be done professionally and considering what is said now, whether the patient is awake or not:

… don't like it when someone is at ease at the expense of the patient… really bothers me when talking about things that do not concern the patient when he/she is present in the operating theatre…. then I will send them out or interrupt the conversation. (#9)

In the surgical team, different nursing interventions are carried out from different professions. OTNs take responsibility that nursing activities not performed at the same time:

… I try to make a sign if it is too chaotic around the patient… it's not good if several persons deal with the patient at the same time, they can do one thing at a time, otherwise it may contribute to negative experiences for the patient. (#1)

OTNs are responsible for maintaining patient dignity and being personally affected when other in the surgical team do not take professional responsibility. All nursing activities must be carried out by agreement in the surgical team.

### 4.8 New understanding

In accordance with Gadamer's hermeneutic, the present findings led to a new understanding of the OTNs' formal external responsibility and personal ethical value for the patient in perioperative practice (see Figure 1). Responsibility was captured by interpreting the OTN's experiences of responsibility for patient care. OTNs have always the patient in mind, which was showed in OTN's formal external responsibility, through ensuring the patient safety and to create a peaceful atmosphere in the operating theatre for the patient's wellbeing. The personal ethical value shows how the OTNs' confirm the patient a person, feel in and be responsive in the situation. This is about seeing, feeling and being responsive, giving confidence and having courage to receive the patient in a mutual community. OTNs' care for the patient is shown by a personal ethical value to always have a look and interest in the patient, to not abandon the patient in a high technological environment and to preserve patient dignity.

## 5 DISCUSSION

In this study, the result shows that the OTN's responsibility included two aspects, a formal external responsibility, to ensure the patient safety and secondly, a personal ethical value to preserved the patient's dignity. According to Eriksson (2007) responsibility involves, having an ethical attitude towards the patient who is in an exposed and vulnerable situation. A new understanding emerged after hermeneutic text interpretation that OTNs always have the patient in mind. There was difficult to find any research in this area.

This result showed that the OTN's formal external responsibility was to control that everything was checked before surgery to ensure patient care takes place, which is the core of perioperative nursing (Rauta, Salanterä, Nivalainen, & Junttila, 2013). The interesting observation in this study was that the OTNs took overall responsibility in the surgical team prior to planning the patient's positioning on the operating table and confirmed the positioning before preoperative preparation. This is because of their knowledge and experience of current surgical technique. Intra‐operatively, the responsibility was to protect the patient's body from pressure and nerve damage by continuously checking the patient's position on the table. They also took the initiative and conducted various relieving exercises, reported when the patient's body was at risk of pressure, checked that the bedding was dry and smooth under the patient and prevented the patient from getting cold (Blomberg, Bisholt, Nilsson, & Lindwall, 2014; Ingvarsdottir & Halldorsdottir, 2017; Kelvered et al., 2012). Another aspect was that the OTN's made sure that the patient was informed and, based on knowledge and experience in perioperative practice, answer the patient's questions when the surgeon was not present. According to AORN Statement Committee (2015) there is a responsibility to provide patients appropriated information to make informed decisions regarding their care.

This study shows that the formal external responsibility meant having preparedness for unforeseen events. Action preparedness described by Björn and Boström (2008) as being in control of the situation and by Mitchell et al. (2011) to have situation awareness and being one step ahead of the surgeon. Sandelin and Gustafsson (2015) emphasize the importance of familiarity of professional skills based on OTNs' previous professional experiences and understanding of each surgeon's different surgical techniques. Gillespie, Chaboyer, Wallis, and Werder (2011) and Stobinski (2015) showed that OTNs with specialist education and an academic degree are better able to recognize subtle cues, demonstrate advanced clinical judgement, problem‐solving and critical thinking, that are associated with increased situation awareness during the perioperative process.

Another result found in the study was the OTN's supervisory function when problems arose related to medical equipment, patient positioning and hygiene or sterility. It is important to prevent postoperative infection (Björn & Boström, 2008; Kelvered et al., 2012) and OTNs took overall responsibility by keeping a watchful eye to ensure that no one in the surgical team compromised sterility (Blomberg, Willassen, von Post, & Lindwall, 2014). The study also revealed OTNs taking responsibility to coordinate and organize work in the surgical team to minimize patient's surgical time. Gillespie et al. (2009a) explains that using a big picture perspective means managing the surgical team to avoid patient delays and cancellations because of lack of availability of material resources and to ensure the right things are done in right order. Another study (Gillespie, Chaboyer, Wallis, Chang, & Werder, 2009b) showed that OTNs are often faced with different challenges to make appropriate decisions in different acute situations and should have the ability to coordinate, negotiate and prioritize according to needs that arise unexpectedly, which also emerged in this study.

Responsibility as a fundamental nursing ethic obtains its determination from caring (Wallinvirta, 2011). In this study, caring was shown when the OTN wanted to confirm the patient as a person to avoid the patient being considered an object. The demands for productivity are high in perioperative nursing and the OTN is a part of the surgical team that performs its work in a minimal amount of time when under pressure. It is a challenge to combine the nursing and caring of the patient with the highly technical aspects of their work (Bull & FitzGerald, 2006; Sørensen et al., 2014). AORN Statement Committee (2015) and perioperative associations in many countries have developed competency standards that recommend that care must be carried out in a person‐centred fashion and planned according to nursing process. According to Stobinski (2015) the standards seem only to be used in the postgraduate program in theatre care. In perioperative practice it seems to be difficult to implement, the organization used their own competence criteria, which affected the OTN's participation in the patient's nursing process and responsibility for patient care remains unclear.

Previous research shows how the patient experienced a mutual community by meeting a known face (Lindwall & von Post, 2009). Lévinas et al. (1988) emphasizes the importance of being there and taking responsibility for the other to fulfil the ethical demand given to him/her. To plan patient care according to the nursing process and OTN's ability to establish a nursing relationship with the patient was to use the ideal model “the perioperative dialogue” which has been shown by research to create continuity in patient care. Patients considered it valuable to receive information and the opportunity to describe their feelings before the surgery and experienced that OTN took personal responsibility for them (Rudolfsson, 2007). Sandelin and Gustafsson (2015) showed that pre‐operative interviews were a prerequisite for safe nursing care contributing to patient safety and when they meet in the operating theatre the patient expressed confidence and a sense of calmness.

Being present meant taking responsibility to reflect on what the patient wants to convey here and now and respect the patient's willingness to participate or not. Martinsen (2012) showed the personal ethical value through being present in the situation and at same time paying attention to what the patient wants to convey with his/her body language, but pointed out that you can never understand another human being without having a relationship. To care for someone is, according to Karlsson, Nyström, and Bergbom (2012) to look and think of the other person who is concerned, with personal involvement. Eriksson (2010) head‐heart‐hand model provides direction in better understanding ethical actions, where the head symbolizes thinking while the heart's motive is good and the hand the esthetical, the beautiful.

The OTN's personal ethical value to preserve patient dignity was to prevent unprofessional behaviour in front of the patient. Willassen, Blomberg, von Post, and Lindwall (2014) found that the OTN was affected when the patient's dignity was not preserved and took responsibility for the humiliation to be stopped. According to Lindwall and von Post (2013), when the patient's dignity is violated and witnessed by an OTN, a value conflict based on personal ethical value is created. Baillie and Ilott (2010) emphasizes to restore patient dignity and pay attention to colleagues, regardless of status, of their behaviour. Ingvarsdottir and Halldorsdottir (2017) means, it is important to speak up even if such comments were not well‐received. Another study showed how OTNs preserved patient dignity through being present for each other and caring for the patient in perioperative practice (Blomberg, Bisholt, et al., 2014). Interestingly, in this study, the OTN was responsible for coordinating different nursing activities to maintain dignity by having the patient in mind. Tiusanen, Junttila, Leinonen, and Salanterä (2010) means that the human‐oriented nursing activities will clarify the responsibility of the nursing care as independent of medical profession. In continued research, focus should be on conflicts of value related to formal and personal responsibility, when there is no opportunity to provide the care that the patient needs.

## 6 LIMITATIONS

Our study has various limitations. For example, a limited number of participants and women were in majority and maybe that can have affected the result. Another limitation was the selection of participants, as nurse managers identified who to ask for participation. It might have contributed to the fact that the most experienced, talented OTNs were asked and the researcher had no control over the selection.

## 7 CONCLUSION

Findings from this study describe that the OTNs experiences a formal external responsibility in perioperative practice, to organized work in the surgical team based on a supervisory function, to ensure the patient's positioning, prevent postoperative infections and minimize the patient′s surgical time. The OTN′s experiences personal ethical responsibility as to confirm the patient as a person and care for the patient. OTNs did not abandon the patient for practical things, but always had the patient in mind and preserved the patient's dignity.

## CONFLICT OF INTEREST

The authors declare that they do not have any conflicts of interest.

## References

[nop2153-bib-0001] Alfredsdottir, H. , & Bjornsdottir, K. (2008). Nursing and patient safety in the operating room. Journal of Advanced Nursing, 61(1), 29–37. 10.1111/j.1365-2648.2007.04462.x 18173734

[nop2153-bib-0002] AORN Statement Committee . (1978). House of delegates – Implications and directions. AORN Journal, 27(6), 1153–1178.

[nop2153-bib-0003] AORN Statement Committee . (2015). Guidelines for perioperative practice. Denver: AORN.

[nop2153-bib-0004] Arakelian, E. , Swenne, C. L. , Lindberg, S. , Rudolfsson, G. , & Vogelsang, A. C. (2016). The meaning of person‐centred care in the perioperative nursing context from the patient's perspective–an integrative review. Journal of Clinical Nursing, 10.1111/jocn.13639 27862496

[nop2153-bib-0005] Baillie, L. , & Ilott, L. (2010). Promoting the dignity of patients in perioperative practice. Journal of Perioperative Practice, 20(8), 278–282. 10.1177/175045891002000802 20860187

[nop2153-bib-0006] Björn, C. , & Boström, E. L. (2008). Theatre nurses' understanding of their work: A phenomenographic study at a hospital theatre. Journal of Advanced Perioperative Care, 3(4), 149–155.

[nop2153-bib-0007] Blomberg, A.‐C. , Bisholt, B. , Nilsson, J. , & Lindwall, L. (2014). Making the invisible visible – operating theatre nurses perceptions of caring in perioperative practice. Scandinavian Journal of Caring Sciences, 29(2), 361–368. 10.1111/scs.12172 25250842

[nop2153-bib-0008] Blomberg, A.‐C. , Willassen, E. , von Post, I. , & Lindwall, L. (2014). Student nurses' experiences of preserved dignity in perioperative practice – Part I. Nursing Ethics, https://doi.org/0969733014542675[pii] 10.1177/0969733014542675 25106458

[nop2153-bib-0009] Bull, R. M. , & FitzGerald, M. (2006). Nursing in a technological environment: Nursing care in the operating room. International Journal of Nursing Practice, 12(1), 3–7. 10.1111/j.1440-172X.2006.00542.x 16403190

[nop2153-bib-0010] Clancy, A. , & Svensson, T. (2007). ‘Faced’ with responsibility: Levinasian ethics and the challenges of responsibility in Norwegian public health nursing. Nursing Philosophy, 8(3), 158–166. 10.1111/j.1466-769X.2007.00311.x 17581243

[nop2153-bib-0011] Eriksson, K. (2007). Vårdvetenskap som akademisk disciplin. Vasa: Åbo Akademi.

[nop2153-bib-0012] Eriksson, K. (2010). Evidence: To see or not to see. Nursing Science Quarterly, 23(4), 275–279. 10.1177/0894318410380271 20870995

[nop2153-bib-0013] Gadamer, H.‐G. (1989). Truth and method (Vol. 2, revis). London: Sheed and Ward.

[nop2153-bib-0014] Gillespie, B. M. , Chaboyer, W. , Wallis, M. , Chang, H.‐Y. A. , & Werder, H. (2009a). Managing the list: OR nurses dual role of coordinator and negotiator. ACORN: Australian College of Operating Room Nurses, 22(1), 5–12.

[nop2153-bib-0015] Gillespie, B. M. , Chaboyer, W. , Wallis, M. , Chang, H.‐Y. A. , & Werder, H. (2009b). Operating theatre nurses' perceptions of competence: A focus group study. Journal of Advanced Nursing, 65(5), 1019–1028. 10.1111/1365-2648.2008.04955.x 19291189

[nop2153-bib-0016] Gillespie, B. M. , Chaboyer, W. , Wallis, M. , & Werder, H. (2011). Education and experience make a difference: Results of a predictor study. AORN Journal, 94(1), 78–90. 10.1016/j.aorn.2010.11.037 21722773

[nop2153-bib-0017] Gillespie, B. M. , & Hamlin, L. (2009). A synthesis of the literature on “competence” as it applies to perioperative nursing. AORN Journal, 90(2), 245–258. 10.1016/j.aorn.2009.07.011 19664414

[nop2153-bib-0018] Gillespie, B. M. , & Pearson, E. (2013). Perceptions of self‐competence in theatre nurses and operating department practitioners. ACORN, 26(1), 29–34.

[nop2153-bib-0019] Helsinki Declaration . (2013). Ethical principels for medical research involving human subject. Available from: http://www.wma.net/en/30publications/10policies/b3/ [Received 25 January 2018].

[nop2153-bib-0020] Ingvarsdottir, E. , & Halldorsdottir, S. (2017). Enhancing patient safety in the operating theatre: From the perspective of experienced operating theatre nurses. Scandinavian Journal of Caring Sciences. 10.1111/scs.12532 28940247

[nop2153-bib-0021] International Council for Nurses . (2013). Code of ethics for nurses. Retrieved from http://www.icn.ch/images/stories/documents/about/icncode_swedish.pdf

[nop2153-bib-0022] Karlsson, M. , Nyström, L. , & Bergbom, I. (2012). To care for the patient: A theory based clinical application research. International Journal of Caring Sciences, 5, 129.

[nop2153-bib-0023] Karnick, P. M. (2016). Power in practice: Moments of reflection. Nursing Science Quarterly, 29(3), 204–205. 10.1177/0894318416647172 27271131

[nop2153-bib-0024] Kelvered, M. , Öhlén, J. , & Gustafsson, B. Å. (2012). Operating theatre nurses experience of patient‐related, intraoperative nursing care. Scandinavian Journal of Caring Sciences, 26(3), 449–457. 10.1111/j.1471-6712.2011.00947.x 22077815

[nop2153-bib-0025] Kvale, S. , Brinkmann, S. , & Torhell, S.‐E. (2009). Den kvalitativa forskningsintervjun, (Vol. 2). Lund: Studentlitteratur.

[nop2153-bib-0026] Lévinas, E. , Nemo, P. , & Contassot, M. G. (1988). Etik och oändlighet: Samtal med Philippe Nemo (Vol. 2). Stockholm; Lund: Symposion.

[nop2153-bib-0027] Lindwall, L. , & von Post, I. (2008). Perioperativ vård – att förena teori och praxis, (Vol. 2). Lund: Studentlitteratur.

[nop2153-bib-0028] Lindwall, L. , & von Post, I. (2009). Continuity created by nurses in the perioperative dialogue – a literature review. Scandinavian Journal of Caring Sciences, 23(2), 395–401. 10.1111/j.1471-6712.2008.00609.x 18785916

[nop2153-bib-0029] Lindwall, L. , & von Post, I. (2013). Preserved and violated dignity in surgical practice – nurses' experiences. Nursing Ethics, 21, 335–346. 10.1177/0969733013498527 24107433

[nop2153-bib-0030] Martinsen, K. (2012). Løgstrup og sygepleien. Aarhus: Klim.

[nop2153-bib-0031] Mitchell, L. , Flin, R. , Yule, S. , Mitchell, J. , Coutts, K. , & Youngson, G. (2011). Thinking ahead of the surgeon. An interview study to identify scrub nurses' non‐technical skills. International Journal of Nursing Studies, 48(7), 818–828. 10.1016/j.ijnurstu.2010.11.005 21190685

[nop2153-bib-0032] Rauta, S. , Salanterä, S. , Nivalainen, J. , & Junttila, K. (2013). Validation of the core elements of perioperative nursing. Journal of Clinical Nursing, 22(9–10), 1391–1399. 10.1111/j.1365-2702.2012.04220.x 23186365

[nop2153-bib-0033] Riley, G. R. , & Manias, E. (2006). Governance in operating room nursing: Nurses' knowledge of individual surgeons. Social Science and Medicine, 62(6), 1541–1551. 10.1016/j.socscimed.2005.08.007 16185800

[nop2153-bib-0034] Rudolfsson, G. (2007). Den perioperativa dialogen: En gemensam värld. Vasa: Åbo Akademi.

[nop2153-bib-0035] Sandelin, A. , & Gustafsson, B. Å. (2015). Operating theatre nurses' experiences of teamwork for safe surgery. Nordic Journal of Nursing Research, 1–7. 10.1177/0107408315591337njn.sagepub.com

[nop2153-bib-0036] SFS . (2003:460). Lag om etikprövning av forskning som avser människor (ändrad 2008:192). Stockholm: Utbildningsdepartementet.

[nop2153-bib-0037] Sjögren, R. (2012). Ansvar In Wiklund Gustin L. & bergbom I. (Eds.), Vårdvetenskapliga begrepp i teori och praktik (pp. 349–360). Lund: Studentlitteratur.

[nop2153-bib-0038] Sørensen, E. E. , Olsen, I. Ø. , Tewes, M. , & Uhrenfeldt, L. (2014). Perioperative nursing in public university hospitals: An ethnography. BMC Nursing, 13(1), 45 10.1186/s12912-014-0045-7 25506263 PMC4264328

[nop2153-bib-0039] Spruce, L. (2013). Bringing back the basics of perioperative nursing care. AORN Journal, 98(5), 438–439. 10.1016/j.aorn.2013.09.001 24209792

[nop2153-bib-0040] Stobinski, J. X. (2015). Certification and Patient Safety. AORN Journal, 101(3), 374–378. 10.1016/j.aorn.2014.11.018 25707730

[nop2153-bib-0041] Tiusanen, T. S. , Junttila, K. , Leinonen, T. , & Salanterä, S. (2010). The validation of AORN recommended practices in finnish perioperative nursing documentation. AORN Journal, 91(2), 236–247. https://doi.org/2048/10.1016/j.aorn.2009.06.027 20152197 10.1016/j.aorn.2009.06.027

[nop2153-bib-0042] von Post, I. (2000). Professionell – at vare etisk i sin hållning (Professional – to be ethical in ones attitude) In Eriksson K., & Lindström U. Å. (Eds.), Gryning I (DawningI) (pp. 161–175). Vasa: Åbo Akademi.

[nop2153-bib-0043] Wallinvirta, E. (2011). Ansvar som klangbotten i vårdandets meningssammanhang. (Diss), Åbo: Åbo Akademi.

[nop2153-bib-0044] Willassen, E. , Blomberg, A.‐C. , von Post, I. , & Lindwall, L. (2014). Student nurses' experiences of undignified caring in perioperative practice – Part II. Nursing Ethics, 22, 688–699. https://doi.org/0969733014542678[pii] 25106457 10.1177/0969733014542678

